# Bone marrow mesenchymal stem cells attenuate the progression of focal segmental glomerulosclerosis in rat models

**DOI:** 10.1186/s12882-018-1137-5

**Published:** 2018-11-22

**Authors:** Ru-chun Yang, Xiao-ling Zhu, Jun Wang, Feng Wan, Hua-qin Zhang, Yi Lin, Xuan-li Tang, Bin Zhu

**Affiliations:** Departmgent of Nephrology (Key laboratory of Zhejiang province, management of kidney disease), Hangzhou Hospital of Traditional Chinese Medicine, Tiyuchang Road 453, Hangzhou, 310007 People’s Republic of China

**Keywords:** BMSC, FSGS, MMP9/TIMP-1, Proinflammatory cytokines, Rat

## Abstract

**Background:**

Focal segmental glomerulosclerosis (FSGS) is the most common glomerular etiology of end-stage kidney disease (ESKD). Increasing evidence has indicated the reparative potential of mesenchymal stem cells (MSCs) in damaged diseased kidneys. However, the effect of bone marrow mesenchymal stem cells (BMSCs) on the FSGS progression remains unclear. This study aimed to investigate the protective effects of BMSCs on FSGS progression.

**Methods:**

A rat model of FSGS was generated via unilateral nephrectomy plus adriamycin injection. Rat BMSCs were isolated and characterized on the basis of their differentiative potential towards adipocytes and osteoblasts and via flow cytometry analysis. Thereafter, rat BMSCs were transplanted into FSGS recipients through the caudal vein. After 8 weeks, 24-h proteinuria, serum creatinine, and urea nitrogen levels were determined. Renal morphology was assessed using a light and transmission electron microscope. MMP9 and TIMP-1 positive cells were detected via immunohistochemical analysis. Expression levels of proinflammatory cytokines IL-6 and TNF-α were examined via RT-PCR.

**Results:**

The isolated adherent cells from the bone marrow of rats were phenotypically and functionally equivalent to typical MSCs. Clinical examination revealed that BMSC transplantation reduced the 24-h urinary protein excretion, and serum creatinine and urea nitrogen levels. Renal morphology was ameliorated in BMSCs-transplanted rats. Mechanistically, BMSC transplantation significantly downregulated TIMP-1 and upregulated MMP9, thereby increasing the renal MMP9/TIMP-1 ratio. Moreover, BMSC transplantation also downregulated IL-6 and TNF-α.

**Conclusions:**

BMSC transplantation can attenuate FSGS progression in a rat model of FSGS, thereby providing a theoretical foundation for the application of autologous BMSCs in clinical FSGS therapy.

## Background

Focal segmental glomerulosclerosis (FSGS) is a heterogeneous disease affecting glomeruli, causing serious scarring [1, 2]. The primary pathological features of FSGS include focal and segmented sclerosis, with proteinuria being the clinical manifestation [3]. FSGS develops rapidly and leads to progressive loss of kidney function. Once the condition becomes uncontrolledable, it may develop into renal failure within 1–2 years, accounting for ~ 15% of cases of end-stage renal disease (ESRD) [4]. Current methods for FSGS treatment primarily rely on hormones and immunosuppressants [5]. However, clinical curative effects are not durable owing to hormone resistance, which occurs in numerous patients [6]. Therefore, it is urgent to develop new treatments for controlling FSGS progression.

Mesenchymal stem cells (MSCs) are connective tissues progenitors, which have emerged as important tools for tissue engineering owing to their multi-potentiality, immune regulation, and multi-factor secretory function [7–10]. In recent years, increasing evidence has indicated the therapeutic potential of MSCs in treating nephropathy [11–14]. Transplantation of bone marrow stem cells (BMSCs) may contribute to glomerular regeneration and repair [15, 16]. In addition, immunosuppression and paracrine function provide insights into the application of MSCs in tissue repair [17, 18]. Briefly, MSCs are anticipated to provide novel insights into FSGS treatment.

This study aimed to investigate the protective effects of BMSCs on FSGS progression in a rat model of FSGS established via unilateral nephrectomy and adriamycin administration. Our results may provide a theoretical basis for the clinical application of MSCs in renal therapeutic interventions.

## Methods

### Animals

Forty clean-grade male Sprague Dawley rats (weighing 160~ 180 g) were purchased from the laboratory animal center of Zhejiang Chinese Medicine University. The rats were housed under standard conditions, as described previously [19]. Animal experiments were performed in accordance with local guidelines for the care of laboratory animals of Animal Experimental Center and were approved by the ethics committee for research on laboratory animal use of the Zhejiang Chinese Medical University.

### Establishment of the FSGS model

All rats were allowed to acclimatize for a week and weighed and assigned randomly to three groups (*n* = 10 rats/group). The rat number was statistics calculated by our preliminary experimental results based on the proteinuria difference between control and model group. To establish the FSGS model, the rats were first subjected to unilateral nephrectomy (left side) on day 1 and then injected 5 mg/kg (on day 7) and 3 mg/kg (on day 28) of adriamycin, dissolved in 0.9% saline at a dilution of 2 mg/mL, in the caudal vein [20]. Meanwhile, the kidneys of the control rats were exposed without dissecting the kidney tissue, followed by layer-by-layer suturing. These rats were then injected saline on day 7 and day 28 through the tail vein after sham operation. Eight weeks post-surgery, the serum and whole kidneys were harvested for biochemical, histological, and molecular analyses, and then animals were euthanized by dislocation of the cervical spine. The urinary protein levels of the rats were quantified before the end of the experiment.

### Isolation and culture of rat BMSCs

Rat BMSCs were isolated in accordance with a previously reported method [21]. Briefly, the cells obtained from the bone marrow were cultured in Iscove’s Modified Dulbecco’s Medium (IMDM; Sigma), supplemented with 10% fetal bovine serum (Hyclone, Rockville, MD, USA), 100 U/mL of penicillin (Gibco BRL, Rockville, MD, USA) and 100 mg/mL streptomycin (Gibco BRL) at 37 °C in 5% CO_2_. After 3d, non-adherent cells and debris were eliminated, and adherent cells were cultured continuously. Upon attaining ~ 90% confluence, cells were digested using 0.25% trypsin-EDTA (Sigma) and then seeded into glass flasks at a cell density of 4 × 10^4^ per cm^2^ as the first passage. The third and fifth passages cells (P3–P5) were used for functional characterization and phenotype analysis. The cultures were maintained by changing the medium every 3 days.

### Characterization of rat BMSCs

#### Differentiation

The cells were characterized on the basis of their mesenchymal lineage differentiation potentials as described previously [22]. For osteogenic differentiation, cells were cultured in IMDM, supplemented with 10 mM β-glycerophosphate, 50 mg/mL ascorbic acid (Sigma), and 10^− 7^ M dexamethasone (Dex), and incubated for 21 d. The differentiated cells were then stained with fresh 0.5% Alizarin Red. Adipogenic differentiation was induced using an adipocyte-inductive medium (IMDM supplemented with 50 μg/mL insulin and 10^− 7^ M Dex). Thereafter, the intracellular lipid droplets were stained with fresh oil Red-O.

#### Flow cytometry (FCM) analysis

The single cell suspension was prepared from the P3–P5 rat BMSCs. After fixing tissues in 4% paraformaldehyde for 15 min, the cells were blocked with 5% normal goat serum for 1 h at 4 °C and incubated with fluorescein-labeled antibodies, including anti-CD29, anti-CD90, anti-MHC-II, and anti-CD45 antibodies. The nonspecific mouse or rabbit IgG served as an isotype control. Afterward, fluorescence signals were determined using a Cytomics FC500 MPL flow cytometer (Beckman Coulter, USA) at 488 nm.

### BMSC transplantation

1 × 10^7^/mL rat BMSCs were transplanted into FSGS recipients through the caudal vein on days 28 and 42, as described previously [23]. Before euthanasia, animals were housed in metabolic cages to collect 24-h urine samples for analysis of urinary protein excretion. Thereafter, the kidneys were harvested for histological analysis and detection of MMP9, TIMP-1, and inflammatory factors. Meanwhile, serums samples were isolated to detect the levels of creatinine and urea nitrogen.

### Histological analysis

Kidney tissues were fixed with 4% paraformaldehyde and embedded in paraffin. For histological analysis of lesions, 3 μm-thick tissue sections were deparaffinized and stained with hematoxylin and eosin (HE) and periodic acid-Schiff (PAS). To calculate focal glomerular sclerosis, 40 to 60 glomeruli from each stained specimen were examined. The degree of sclerosis in each glomerulus was subjectively graded on a scale of 0 to 4 as follows: Grade 0, no change; Grade 1, sclerotic area less than or equal to 25% of the glomerulus or the presence of distinct adhesion between the capillary tuft and Bowman’s capsule; Grade 2, sclerosis of 25 to 50% of the total glomerular area; Grade 3, sclerosis of 50 to 75% of the total glomerular area; and Grade 4, sclerosis of more than 75% of the glomerulus. The glomerular sclerosis index (GSI) was calculated by using the following formula as previously reported [24]:

GSI =$\frac{\left(1\times \mathrm{N}1\right)+\left(2\times \mathrm{N}2\right)+\left(3\times \mathrm{N}3\right)+\left(4\times \mathrm{N}4\right)}{\left(\mathrm{N}0+\mathrm{N}1+\mathrm{N}2+\mathrm{N}3+\mathrm{N}4\right)}$, where N is the number of glomeruli at each grade of sclerosis.

For immunohistochemistry, paraffin-embedded kidney sections were blocked with 5% goat serum at 37 °C for 1 h, and then stained with anti-MMP-9 and anti-TIMP-1 antibodies (1:500 and 1:50, respectively; Boster Biological Technology Co., Ltd., Wuhan) in accordance with the manufacturers’ instructions. Thereafter, kidney tissues were incubated with HRP-conjugated anti-rabbit/mouse IgG Ab (1,8000) at 37 °C for 1 h. Finally, the slides were incubated with DAB peroxidase substrate (Sigma). The staining results were obtained via light microscope (Olympus BX51).

### Transmission electron microscopy (TEM)

After fixation in 2.5% glutaraldehyde for at least 3 h, the kidney tissue (~ 1 mm^3^ in size) was rinsed in 0.1 M PBS thrice, post-fixed with 1% osmium tetroxide for 1 h, dehydrated in graded acetone and embedded in graded Epon 812. Thin sections (80–100 nm) were cut and stained with uranyl acetate (2%) and lead citrate before observation under a JEM-1400 transmission electron microscope (JEOL, Japan).

### Real-time quantitative PCR

Total RNA was extracted from kidney tissue using TRIzol reagent (Invitrogen, USA) and was reverse-transcribed to cDNA, using a PrimescriptTM RT reagent kit (TaKaRa, China). Thereafter, expression levels of IL-6 and TNF-α were quantified via real-time PCR using Applied Biosystems 7500 Fast real-time PCR system (Thermo Fisher Scientific, USA) and detection system software, using primers shown in Table 1. Relative mRNA expression levels were normalized to those of GAPDH via the 2^−ΔΔCT^ method. Each PCR experiment was performed in triplicate and repeated independently at least thrice.

**Table 1:** Primers used for gene expression analyses

Primer Name	Sequence (5′ to 3′)	Application
Rat-IL-6-F	CCTTCTTGGGACTGATGT	Real-time RT-PCR
Rat-IL-6-R	TCCAGGTAGAAACGGAAC	Real-time RT-PCR
Rat-TNF-α-F	GGCGTGTTCATCCGTTCT	Real-time RT-PCR
Rat-TNF-α-R	CCACTACTTCAGCGTCTCG	Real-time RT-PCR
Rat-GAPDH-F	ACCACAGTCCATGCCATCAC	Real-time RT-PCR
Rat-GAPDH-R	TCCACCACCCTGTTGCTGTA	Real-time RT-PCR

### Statistical analysis

All values are presented as the mean ± SD values. The data shown were analyzed for significance via Student’s *t*-tests or One-Way ANOVA using SPSS17.0 software. A *p*-value less than 0.05 or 0.01 indicated statistical significance. Besides, the investigators for group allocation and data analysis in our experiments were both blinded.

## Results

### Isolation and characterization of rat BMSCs

**Fig. 1:**
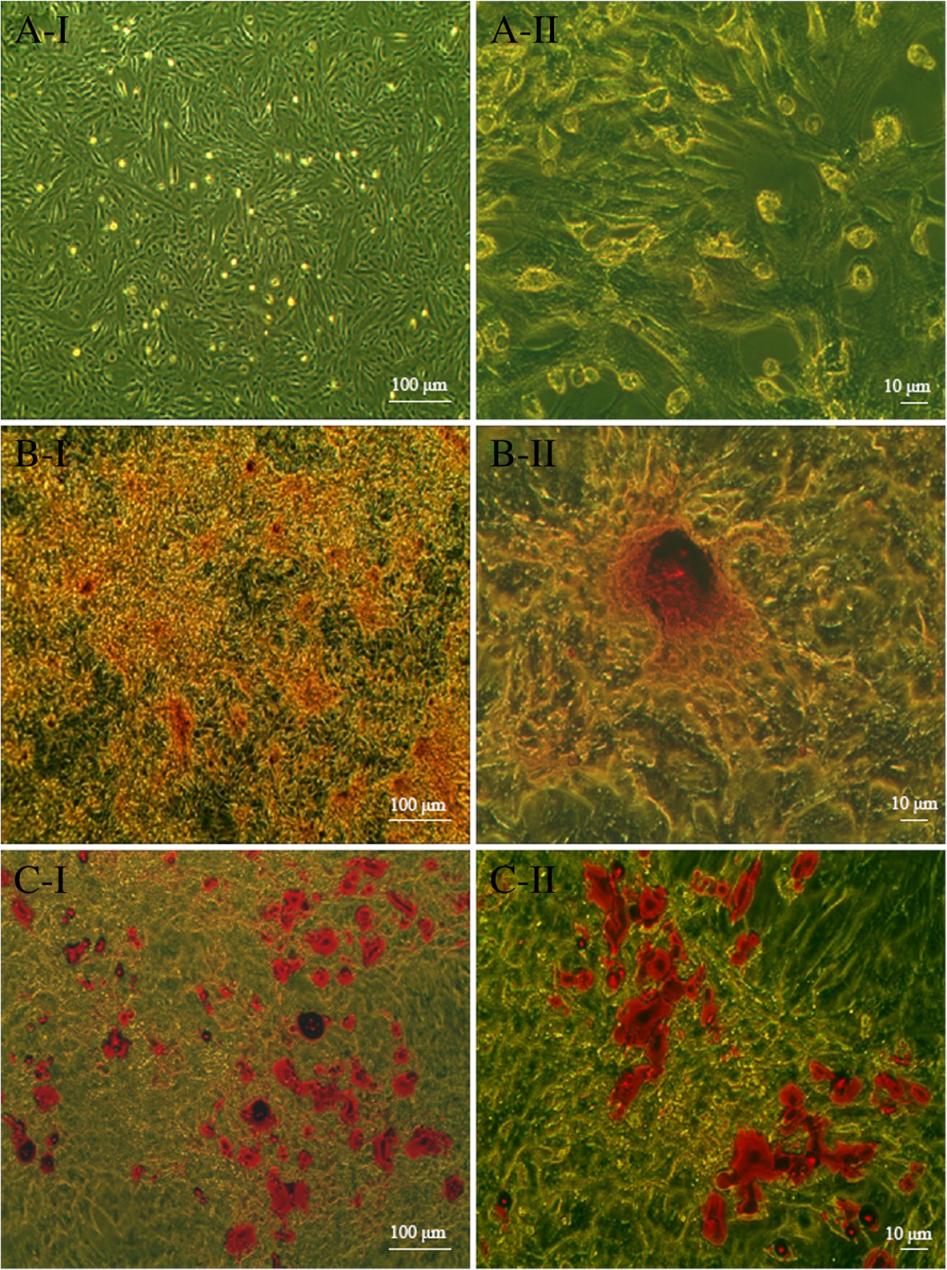
Characterization of rat BMSCs. **a** Morphological observation of the adherent P3 cells under an inverted microscope. **b** Adipogenic differentiation of BMSCs: BMSCs were cultured for 21 d alternately in adipogenic induction medium. Lipid droplets were visualized by Oil Red O staining. **c** Osteogenic differentiation of BMSCs: cells were cultured for 21 d with osteogenic induction medium, and calcium deposits were visualized by Alizarin Red staining. **a-c** I: original magnification × 40; **a-c**: II, original magnification × 200

**Fig. 2:**
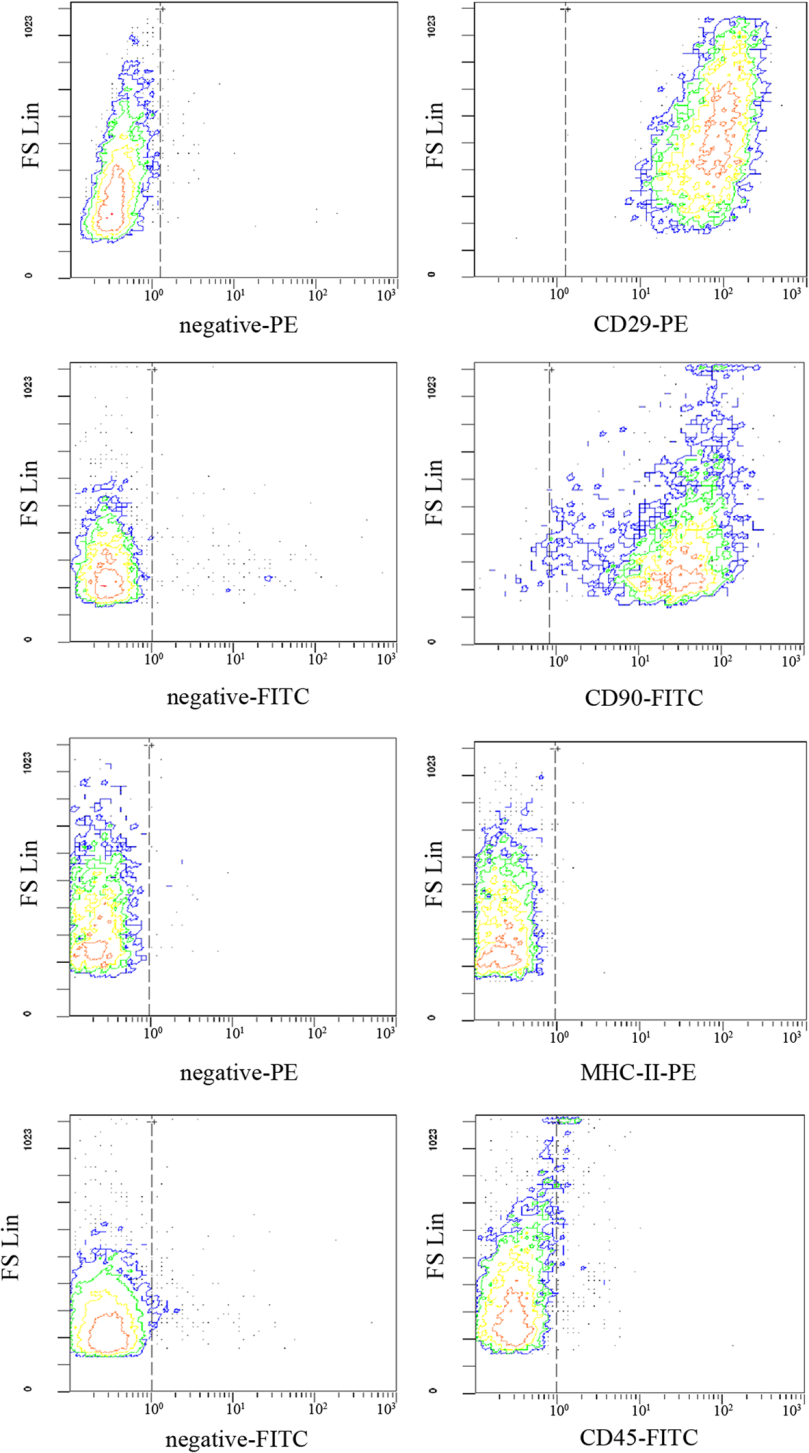
Immunophenotypic analyse of BMSCs by FCM. Single cell suspensions of BMSCs were stained with CD29, CD90, MHC-II, and CD45, respectively. Unrelated IgG was used as negative control

Rat BMSCs were harvested, purified, and cultured in vitro. Majority of the isolated cells displayed spindle-like morphology. After more than 3 passages in culture, they tended to be morphologically more heterogeneous (Fig. 1a). For further identification, the mesodermal differentiation potential of these cells was evaluated. The cells were induced to differentiate into adipocytes and osteocytes in vitro, using adipogenic and oseteogenic induction media, respectively. Following 3 weeks of adipogenic induction, these cells exhibited lipid-laden adipocyte phenotype, indicated by a positive result on Oil Red ‘O’ staining (Fig. 1b). Similarly, when induced with osteogenic induction medium for 2 to 3 weeks, they also displayed osteogenesis upon staining with Alizarin Red for calcium deposits (Fig. 1c). Finally, flow cytometry analysis was performed to analyze the phenotypic characteristics of the isolated cells. The isolated cells were CD29- and CD90-positive, and negative for MHC-II and CD45 (Fig. 2). Collectively, these results strongly indicate that the isolated adherent cells were phenotypically and functionally equivalent to typical MSCs.

### BMSC transplantation reduced urinary protein excretion of FSGS rats

**Fig. 3:**
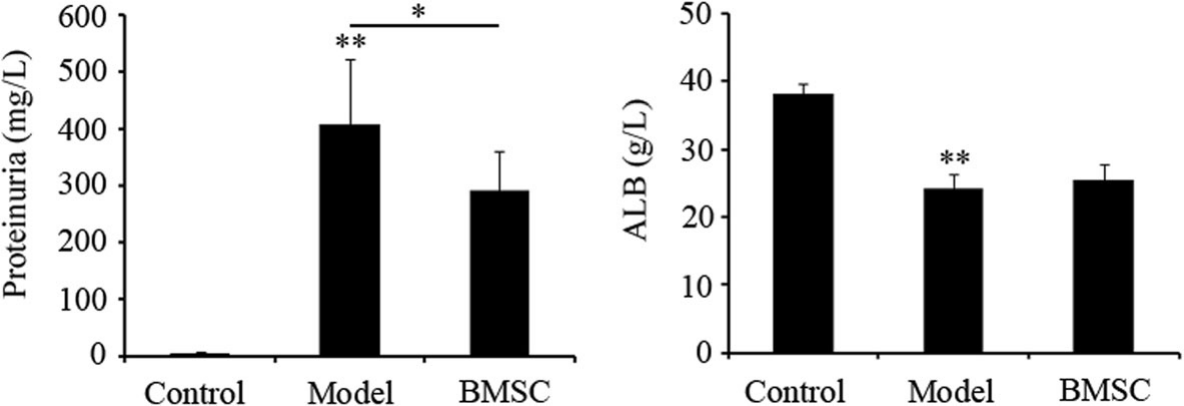
Evaluation of proteinuria concentration. Twenty four-hour urine was collected from rats of control, model and BMSC-treated groups (Control, Model, and BMSC), and total urinary protein was measured by a chemistry analyzer (HITACHI 7180). Data (*n* = 10) are presented as the mean ± SD, *p < 0.05, vs. BMSC and model groups; **p < 0.01, vs. model and control groups

Through unilateral nephrectomy plus adriamycin injection, the FSGS rat model was successfully established, which subsequently lead to the emergence of increased proteinuria after 6 weeks (Fig. 3, *p < 0.01*), as a typical clinical feature of FSGS. Meanwhile, the serum albumin (ALB) in the model group decreased significantly compared with the control group (Fig. 3, *p < 0.01*). Although the ALB level has no obvious improvement, the 24-h urinary protein excretion in BMSC-treated group of rats was much lower than in the model group at 6 weeks (Fig. 3, *p < 0.05*), indicating the attenuation of renal injury progression to some extent by BMSC transplantation.

### BMSC transplantation improve the kidney function of FSGS rats

**Fig. 4:**
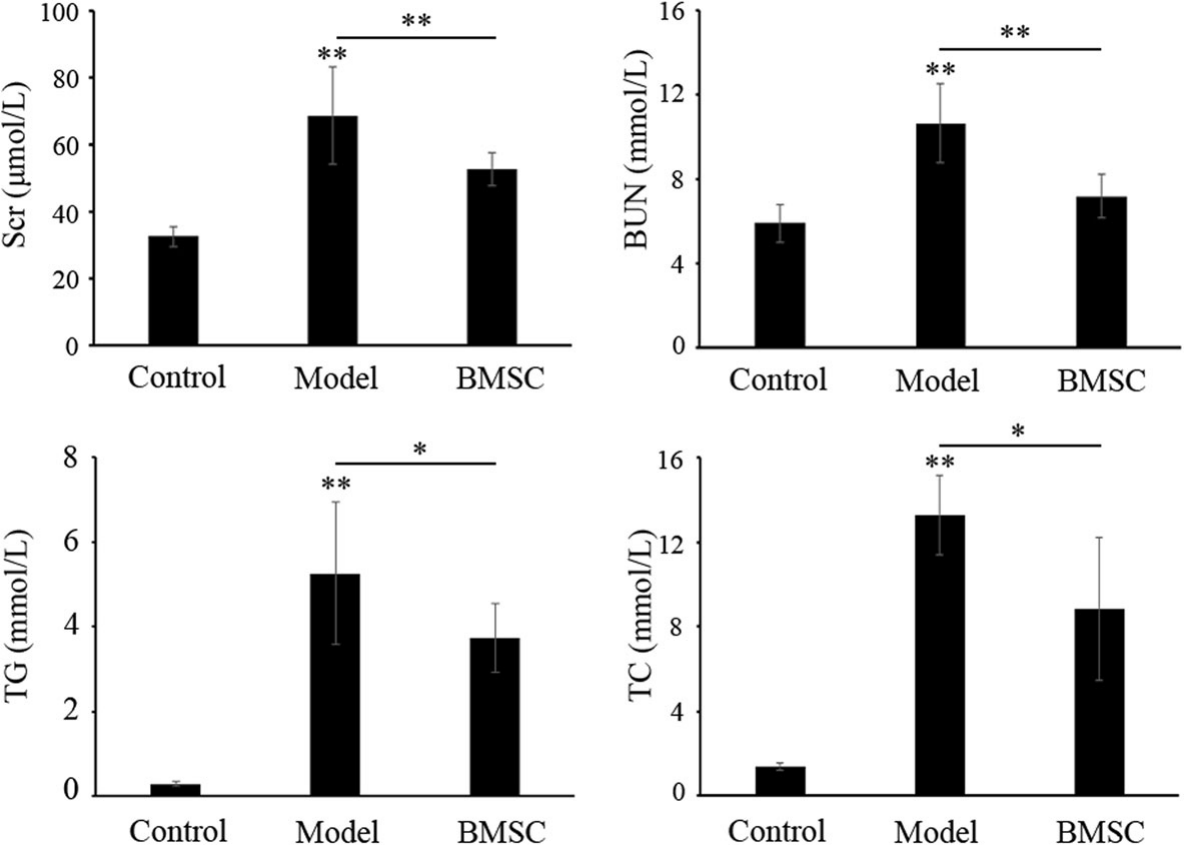
Evaluation of serum creatinine and urea nitrogen concentration. Serum creatinine and urea nitrogen were collected and measured by a chemistry analyzer (HITACHI 7180). Data (*n* = 10) are presented as the mean ± SD, **p < 0.01

To evaluate renal dysfunction, changes in serum creatinine (Scr) and urea nitrogen (BUN) were initially examined. Results showed that while the control rats had normal levels of Scr (32.6 ± 2.88 μmol/L) and BUN (5.9 ± 0.89 mmol/L), the model group had increased levels of both (68.7 ± 14.38 μmol/L and 10.65 ± 1.88 mmol/L, respectively; Fig. 4, *p* < *0.01*). Compared to that in the model group, Scr and BUN levels in BMSC transplantation group were significantly lower (52.9 ± 4.95 μmol/L and 7.19 ± 1.03 mmol/L, respectively, *p < 0.01*). Meanwhile, the cholesterol (TC) and triglycerides (TG) levels were also examined. Expectedly, they showed similar tendency with that of Scr and BUN (Fig. 4). These results suggested that BMSC treatment could improve kidney function.

### BMSC transplantation affected renal morphology

**Fig. 5:**
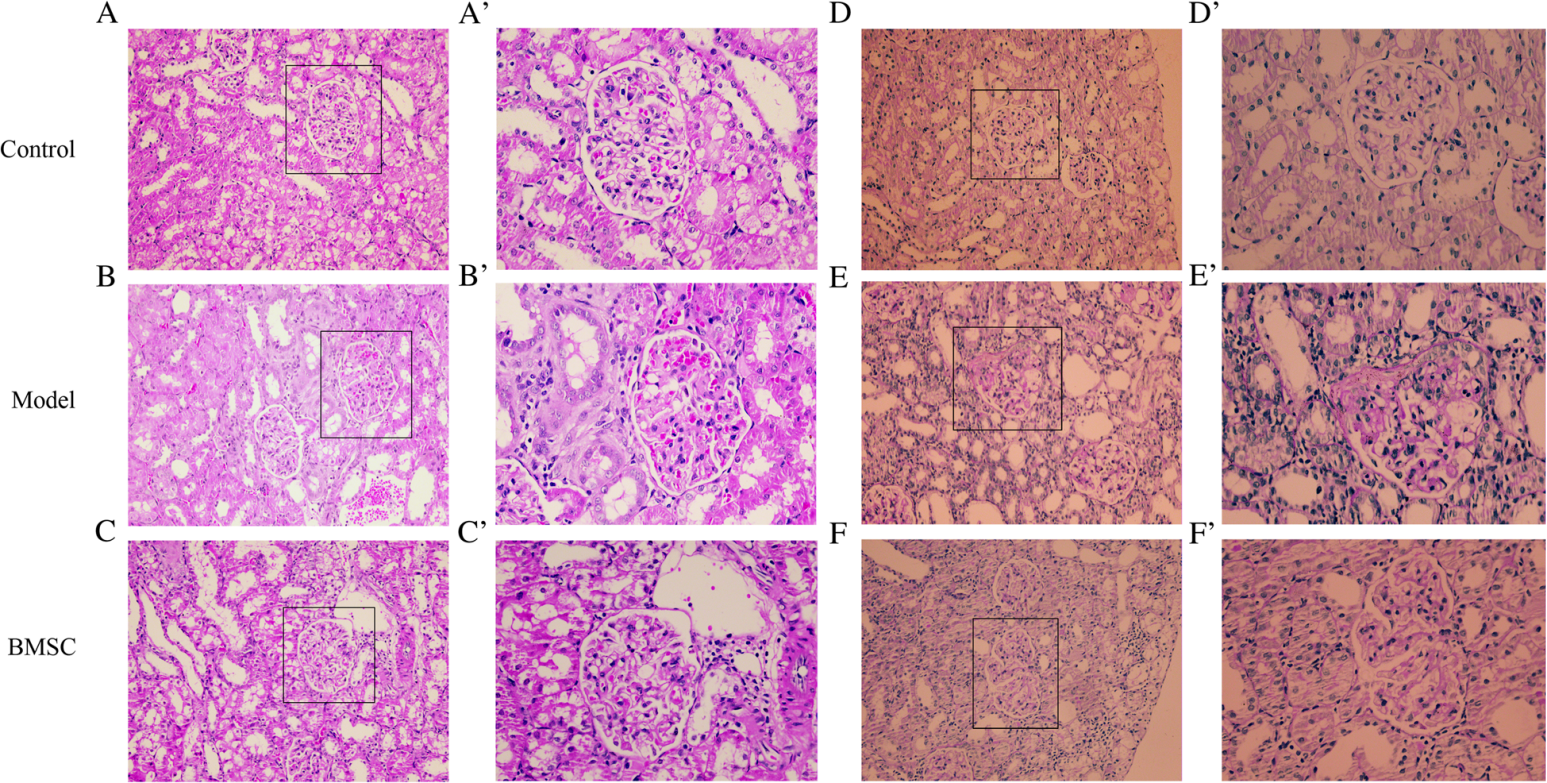
Analysis of renal morphology by light microscopy. Histological analysis of kidney from the model group revealed focal glomerular sclerosis in some glomerular and interstitial lesions. The lesions were significantly alleviated in the BMSCs treated group. The left panel (A-C) represents HE staining and the right panel (E-F) represents PAS staining (original magnification × 200). *n* = 10 animals in each group. Higher magnifications of the glomerulus marked by solid black square in (A-C) and (E-F) were shown in (A´-C´) and (E´-F´)

**Fig. 6:**
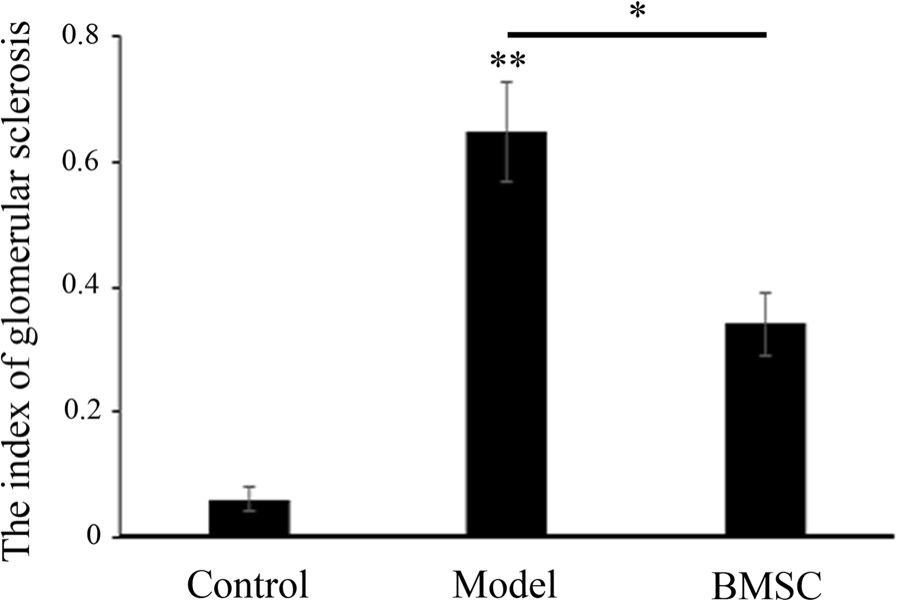
The glomerular sclerosis index in each group. *p < 0.05, **p < 0.01

Pathological lesions in the kidney tissues were further examined by histological analysis. Light microscopy results of HE and PAS staining showed obvious pathological changes in rat kidneys from the model group, characterized by focal glomerular sclerosis in some glomerular and interstitial lesion (Fig. 5). In the BMSC-treated group, such pathological damages were much less compared to that in the model group, whereas no pathological change was found in kidneys from the control group (Fig. 5). Correspondingly, the glomerular sclerosis index (GSI) was higher in the model group than that of the control group (Fig. 6, *p < 0.01*). However, the BMSC-treated group significantly decreased the GSI compared to that in the model group (Fig. 6, *p < 0.05*).

**Fig. 7:**
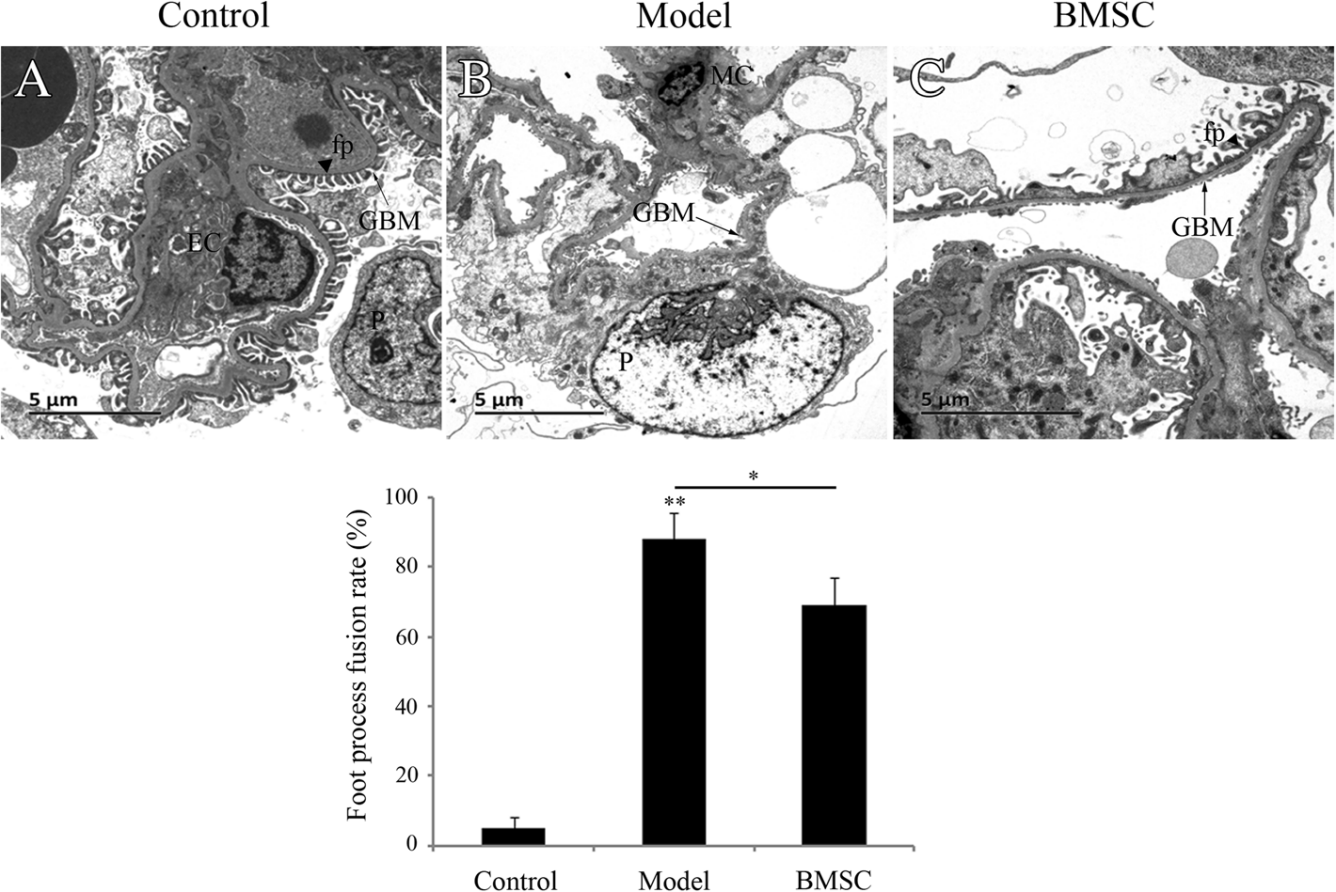
Analysis of renal morphology by TEM. **a** The foot processes in the control group were intact. **b** The foot processes diffusely effaced, became flat and fused and even disappeared in the model group. The lesions were significantly alleviated in the BMSC treated rats compared to that in the model group (**c**). GBM, glomeruler basement membrane (black arrow); EC, endothelial cells; MC, mesangial cells; P, podocyte; fp, podocyte foot process (black triangle). Scale bar 5 μm (bottom left). Original magnification × 5000, *n* = 10 animals in each group

Correspondingly, TEM analysis showed intact glomeruli and clear foot processes on the visceral surface of the renal glomerular epithelial cells in the control rats. However, the foot processes diffusely effaced, became flat and fused and even disappeared in the model group (Fig. 7). The foot process fusion rate (88% ± 7.55%) increased in the model group compared with the control group (5.2% ± 3.11%). Besides, the glomerular basement membrane was thickened and collapsed (Fig. 7). In the BMSC-treated group, the lesions were significantly alleviated and the foot fusion rate reduced compared with the model group (69% ± 7.94%). These results indicated BMSC transplantation can improve renal morphology.

### BMSC transplantation improved the renal MMP/TIMP imbalance

**Fig. 8:**
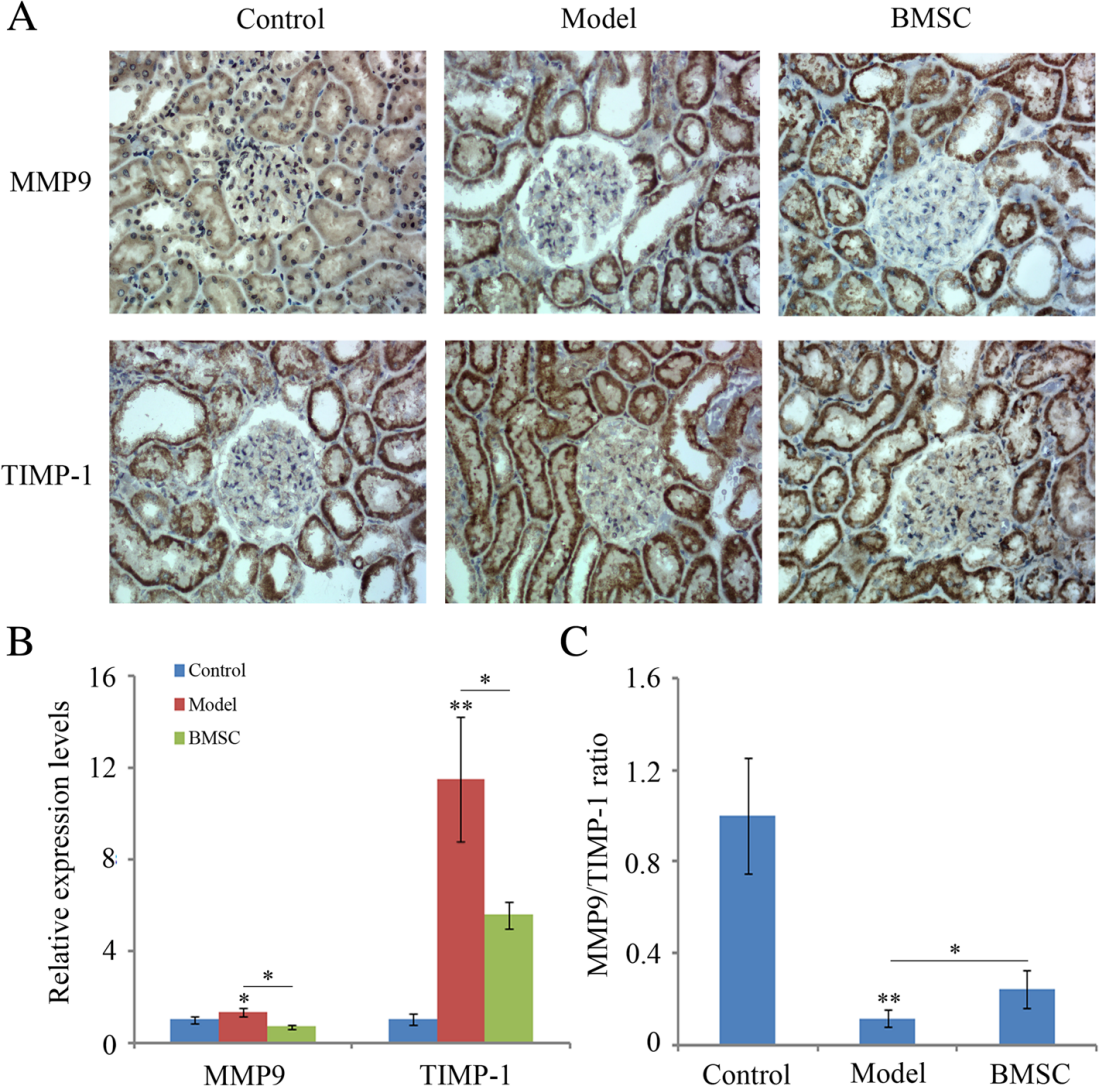
Immunohistochemical analyse of renal MMP9 and TIMP-1. **a** Paraffin sections of kidney from control, model and BMSC-treated groups (Control, Model and BMSC respectively) were stained with MMP9 and TIMP-1. The staining results were observed under a light microscope, original magnification × 400. **b** The expression of MMP9 and TIMP-1 were determined by RT-PCR. Quantitative data are provided as the mean ± SD. *p < 0.05, **p < 0.01. **c** The MMP9/TIMP-1 ratio was further calculated. The statistical data (*n* = 10) are given as the mean ± SD, **p < 0.01, *p < 0.05

Matrix metalloproteinases (MMPs) are reported as important markers of deleterious remodeling associated with the progression of renal diseases [25]. The relative balance between MMP and its tissue inhibitor TIMP is considered to determine the rate of extracellular matrix (ECM) turnover. Therefore, we evaluated the expression of MMP9 and TIMP-1 in rats from different groups. Immunohistochemical results showed MMP9 to be abundant in renal tubule and TIMP-1 mainly localized to the renal tubular epithelium, interstitium, and glomerular mesangial region (Fig. 8a). Moreover, RT-PCR results indicated the mRNA expression level of MMP9 and TIMP-1 were both much higher than that in the control group (Fig. 8b, *p < 0.01 or p < 0.05*), resulting the MMP9/TIMP-1 ratio decreased significantly (Fig. 8c, *p < 0.01*). Compared to that in the model group, BMSC transplantation significantly decreased MMP9 and TIMP-1 expression (Fig. 8b, *p < 0.01*). Besides, renal MMP9/TIMP-1 ratio was also elevated, which may lead to the decrease in ECM accumulation. Taken together, these results suggested that BMSC transplantation may improve renal MMP/TIMP-1 imbalance.

### BMSC transplantation suppressed the expression of renal inflammatory factors in FSGS rats

**Fig. 9:**
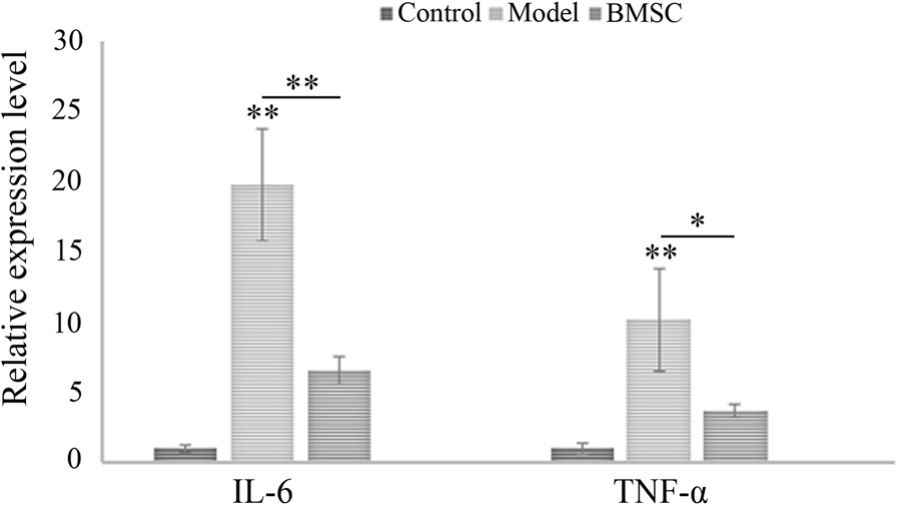
Analysis of the expression of renal inflammatory factors by RT-PCR. Transcription of inflammatory factors, including IL-6 and TNF-α was detected by RT-PCR, and mRNA expression levels were normalized to that of GAPDH. Quantitative data (*n* = 10) are provided as the mean ± SD. *p < 0.05, **p < 0.01

Considering the pathogenic role of inflammation in the development of renal fibrosis, expression of several important inflammatory factors were further evaluated in our study. We found that expression of IL-6 and TNF-α remarkably increased in the model group compared to that in the control group. However, the expression decreased significantly in BMSC-treated group, indicating inflammatory inhibition upon BMSC transplantation (Fig. 9).

## Discussion

FSGS was first described in kidney biopsy of adults with nephritic syndrome in 1925 [26]. It has since been considered a lesion, rather than a disease, with morphologic variations including tip, perihilar, cellular, collapsing, and not-otherwise-specified features [27]. FSGS is a clinicopathologic entity that is characterized frequently by steroid-resistant nephrotic syndrome and rapid progression to end-stage kidney disease (ESKD) in majority of affected individuals [4]. The therapeutic agents, currently available for the treatment of FSGS, are not very effective and only few patients have shown complete remission. Therefore, to seek a safer and more effective method to revert FSGS progression is an imminent challenge.

In recent years, positive effects of MSCs on different models of chronic kidney diseases have been reported, mainly as improvements in proteinuria, glomerulosclerosis, macrophage infiltration, renal fibrosis, renal filtration, and plasma creatinine [12, 14, 28–31]. In our present study, the effect of BMSCs on FSGS was evaluated by a series of assays in a rat model. Adriamycin nephropathy is a common kidney disease model, that resembles FSGS in its chronic phase [20]. Our study found that unilateral nephrectomy and adriamycin injection in rats had massive proteinuria after 6 weeks, hence coinciding with the clinical manifestations of FSGS. Biochemical and histological changes suggested that adriamycin nephropathy was successfully established in our experimental preparation. After BMSC transplantation, the progression of FSGS was prevented. Initially, 24-h urinary protein excretion decreased significantly in BMSC-treated group of rats, indicating attenuation of renal injury progression by BMSC transplantation. Biochemical analysis results showed that the rats with BMSC transplantation had much lower serum creatinine and urea nitrogen levels, which in turn suggested recovery of the injured renal function. Immunohistochemical and RT-PCR results showed increase of MMP9 and decrease of its tissue inhibitor TIMP-1, thereby leading to an enhanced ratio of MMP/TIMP and hence degradation of extracellular matrix (ECM), which may be important for preventing renal fibrosis. Finally, our study found renal IL-6 and TNF-α to be much higher in the model group than in the control group, hence suggesting accompanied by inflammatory responses in the FSGS rat. When the BMSCs were transplanted, the expression levels of these inflammatory factors were suppressed. Based on previous studies, we speculated that BMSC transplantation may attenuate the progression of FSGS by immune-regulation.

Taken together, we present the results of a preliminary study of application of BMSCs on FSGS treatment in a rat model. The beneficial effect of BMSCs might be mediated by increasing the MMP/TIMP ratio, and down-regulating the pro-inflammatory cytokines to decrease inflammatory cell infiltration in the renal interstitium. Our study provides an experimental basis for application of BMSC therapy in renal diseases. However, the underlying mechanisms for BMSCs in FSGS need to be addressed in future.

## Conclusions

We established the FSGS rat model through unilateral nephrectomy and adriamycin injection, and investigated the effect of BMSCs on FSGS treatment in this paper. The main indices include 24-h urinary protein, serum creatinine, urea nitrogen, and renal morphology. In terms of mechanism, BMSCs may relieve FSGS by improving the imbalance of renal MMP/TIMP and suppressing the expression of renal inflammatory factors IL-6 and TNF-α. The precise mechanism involved is still under investigation.

## Abbreviations

(ECM): Extracellular matrix
BMSC: Bone mesenchymal stem cell
ESKD: End-stage kidney disease
FCM: Flow cytometry
FSGS: Focal segmental glomerulosclerosis
HE: Hematoxylin and eosin
IMDM: Iscove’s Modified Dulbecco’s Medium
MMP: Matrix metalloproteinase
MSCs: Mesenchymal stem cells
PAS: Periodic acid-Schiff
TIMP-1: Tissue inhibitor of matrix metalloprotease-1
